# The involvement of interleukin-1 and interleukin-4 in the response of human annulus fibrosus cells to cyclic tensile strain: an altered mechanotransduction pathway with degeneration

**DOI:** 10.1186/ar3229

**Published:** 2011-01-28

**Authors:** Hamish TJ Gilbert, Judith A Hoyland, Anthony J Freemont, Sarah J Millward-Sadler

**Affiliations:** 1 Regenerative Medicine, School of Biomedicine, Faculty of Medical and Human Sciences, University of Manchester, Stopford Building, Oxford Road, Manchester, M13 9PL, UK

## Abstract

**Introduction:**

Recent evidence suggests that intervertebral disc (IVD) cells derived from degenerative tissue are unable to respond to physiologically relevant mechanical stimuli in the 'normal' anabolic manner, but instead respond by increasing matrix catabolism. Understanding the nature of the biological processes which allow disc cells to sense and respond to mechanical stimuli (a process termed 'mechanotransduction') is important to ascertain whether these signalling pathways differ with disease. The aim here was to investigate the involvement of interleukin (IL)-1 and IL-4 in the response of annulus fibrosus (AF) cells derived from nondegenerative and degenerative tissue to cyclic tensile strain to determine whether cytokine involvement differed with IVD degeneration.

**Methods:**

AF cells were isolated from nondegenerative and degenerative human IVDs, expanded in monolayers and cyclically strained in the presence or absence of the cytokine inhibitors IL-1 receptor antagonist (IL-1Ra) or IL-4 receptor antibody (IL-4RAb) with 10% strain at 1.0 Hz for 20 minutes using a Flexcell strain device. Total RNA was extracted from the cells at time points of baseline control and 1 or 24 hours poststimulation. Quantitative real-time polymerase chain reaction was used to analyse the gene expression of matrix proteins (aggrecan and type I collagen) and enzymes (matrix metalloproteinase 3 (*MMP3*) and a disintegrin and metalloproteinase with a thrombospondin type 1 motif 4 (*ADAMTS4*)).

**Results:**

Expression of catabolic genes (*MMP3 *and *ADAMTS4*) decreased in AF cells derived from nondegenerative tissue in response to 1.0-Hz stimulation, and this decrease in gene expression was inhibited or increased following pretreatment of cells with IL-1Ra or IL-4RAb respectively. Treatment of AF cells derived from degenerative tissue with an identical stimulus (1.0-Hz) resulted in reduced anabolic gene expression (aggrecan and type I collagen), with IL-1Ra or IL-4RAb pretreatment having no effect.

**Conclusions:**

Both IL-1 and IL-4 are involved in the response of AF cells derived from nondegenerative tissue to 1.0-Hz cyclic tensile strain. Interestingly, the altered response observed at 1.0-Hz in AF cells from degenerative tissue appears to be independent of either cytokine, suggesting an alternative mechanotransduction pathway in operation.

## Introduction

The intervertebral disc (IVD), comprising a central gelatinous nucleus pulposus (NP) and the peripheral collagenous annulus fibrosus (AF), is a fibrocartilage pad which functions to provide stability to the spine while enabling flexibility through all planes. *In vivo *the disc is exposed to a range of dynamic mechanical stimuli with physiological ranges of force known to lead to matrix homeostasis in healthy disc cells [1-7], while nonphysiological magnitudes, frequencies and durations of force result in matrix catabolism [5,8-16]. Degenerative disc disease (DDD), characterised by the deterioration and degradation of disc matrix, has been shown to affect disc cell mechanobiology, leading to the inability of disc cells to respond to physiological loads in the normal anabolic manner. For example, Le Maitre *et al. *[17] found that human NP cells derived from degenerative IVD tissue (unlike human NP cells derived from nondegenerative tissue) were unable to respond to hydrostatic pressures (HP). Furthermore, we have recently shown that the reduced catabolic response of human AF cells derived from nondegenerative tissue exposed to 1.0-Hz cyclic tensile strain (CTS) is aberrant in degenerative human AF cells, resulting in an overall catabolic response [18]. Importantly, this shift from an overall anabolic to a predominantly catabolic response could lead to further degradation of the extracellular matrix (ECM) and ultimately to the progression of DDD.

Cellular mechanotransduction is defined as the process by which a cell is able to sense a mechanical or physical force, convert it into an intracellular biochemical signal and thus alter cellular metabolism to regulate ECM homeostasis. A variety of intracellular signalling proteins and kinases have been implicated in the mechanotransduction pathways of numerous cell types. These include activation of stretch-activated and calcium-sensitive ion channels [19], protein tyrosine phosphorylation [20], activation of protein kinase C (PKC) [21] and initiation of mitogen-activated protein kinase (MAPK) pathways [22]. Activation of these pathways can, in turn, lead to the synthesis of important regulatory molecules involved in regulating tissue structure and function. These include the synthesis of proteoglycan by IVD cells [16], the release of nitric oxide (NO) and prostaglandins by tenocytes [23], the production of platelet-derived growth factor by smooth muscle cells [24], and the release of cytokines by chondrocytes [25,26].

Although mechanical stimulation is recognised as an important regulatory factor in IVD biology and ECM homeostasis [27,28], studies in which IVD cell mechanotransduction pathways have been investigated are limited. This is surprising, as it is likely that the aberrant response observed in disc cells derived from degenerative tissue exposed to mechanical stimulation is due to alterations in the mechanotransduction pathway active in these cells. It follows, therefore, that if the mechanotransduction pathway of disc cells derived from degenerative tissue is altered, defining the signalling pathway could lead to the discovery of novel therapeutic targets for the prevention and/or treatment of DDD.

Using arginine-glycine-aspartic acid (RGD) function-blocking peptides, Le Maitre *et al. *[29] showed that the compression-induced decrease in aggrecan gene expression observed in human NP cells occurs through the involvement of integrins in NP cells derived from nondegenerative but not degenerative tissue, suggesting an altered mechanotransduction pathway in operation. Liu *et al. *[30] found that proteoglycan synthesis was stimulated and inhibited in a heterogeneous population of human AF and NP cells exposed to low and high HP, respectively, with NO production levels inversely correlated with proteoglycan synthesis. Furthermore, this HP-stimulated alteration in disc cell proteoglycan synthesis could be prevented following pretreatment of cells with NO synthase inhibitors, suggesting NO as a mechanosensitive soluble mediator [30]. Similarly, using rabbit AF cells, Rannou *et al. *[31] found that the 5% CTS-stimulated reduction in proteoglycan synthesis occurs in parallel with increased NO production and that, after the addition of NO synthase inhibitors, this CTS-stimulated decrease in proteoglycan synthesis was abolished.

In addition to NO, other soluble mediators, including prostaglandins and cytokines, have also been implicated in the mechanoresponse of cells from other tissues, including bone cells [32-35], endothelial cells [36,37], tendon cells [23,38] and chondrocytes [25,26,39,40]. Interestingly, a number of cytokines have been implicated in mechanotransduction pathways in articular chondrocytes, a cell type which shares many similarities with cells of the IVD [41], although to date their involvement in IVD cell mechanotransduction has not been elucidated. Mohtai *et al. *[25] reported increased expression of IL-6 at the gene and protein levels in human chondrocytes following exposure to fluid-induced shear strains. Millward-Sadler *et al. *[26] found that the hyperpolarisation response of chondrocytes derived from nonosteoarthritic cartilage stimulated with 0.33-Hz CTS was abolished when the cells were first treated with neutralising antibodies to IL-4 and its receptors, while pretreatment with neutralising antibodies to IL-1 β had no effect on this mechanoresponse. Interestingly, when the same experiment was conducted using chondrocytes from osteoarthritic cartilage, the electrophysiological response to 0.33-Hz CTS was altered to a depolarisation response and was inhibited after pretreatment with neutralising antibodies to both IL-1 β and IL-4. Such data demonstrate the involvement of these cytokines in this mechanoresponse, and suggests differential mechanotransduction pathways in operation between chondrocytes isolated from nonosteoarthritic and osteoarthritic cartilage [40].

IL-1 has not previously been investigated as a signalling molecule in IVD cell mechanotransduction, although it is well recognised as an important inflammatory mediator in disc biology. Associated with the pathogenesis of DDD, IL-1 stimulates catabolic gene and protein expression and is found in greater amounts with increasing severity of disc degeneration [42,43]. In addition to this, IL-1 β-induced increased catabolism in rat AF cells has been shown to be partially inhibited by CTS treatment, suggesting a potential interaction between these two signalling pathways [44]. Although IL-4 has previously been described in the IVD and has been shown to increase with degeneration, its role in disc biology remains unknown [45,46]. However, in chondrocyte biology, in addition to its reported role in mechanotransduction, IL-4 functions as a chondroprotective cytokine capable of inhibiting the expression of inflammatory mediators [47] and reducing catabolic gene and protein expression [48], and has been shown to protect against mechanically stimulated increased matrix metalloproteinase 13 (*MMP13*) gene expression in cyclically strained rat chondrocytes [49].

To date, no studies have investigated the role of these cytokines in the mechanotransduction pathway of mechanically stimulated IVD cells. Therefore, the aim of this study was to investigate the involvement of IL-1 and IL-4 in human AF cell mechanotransduction and to ascertain whether the previously observed differences in cellular responses between AF cells derived from nondegenerative and degenerative tissue [18] are due to an alteration in the mechanotransduction pathways.

## Materials and methods

### IVD tissue

Human IVD tissue was collected from patients undergoing lumbar spinal surgery for DDD or from cadavers (within 18 hours of death) with the consent of the patients or their relatives and the approval of the Central Manchester, Bury, Rochdale, Salford and Trafford Research ethics committees. Tissue was processed for cell extraction, and representative samples of all tissues containing intact AF and NP regions were formalin-fixed and paraffin-embedded, and sections were histologically graded as previously reported [41]. Graded tissue was given a score between 0 and 12, with 0 to 3 being classified as nondegenerative, 4 to 7 being classified as mildly degenerative and 8 to 12 being classified as severely degenerative. Nondegenerative IVDs were collected from three cadavers (mean donor age, 47 years; age range, 37 to 57 years), and histologically degenerative IVDs were collected from two patients who had undergone surgery for DDD and from one cadaver (mean donor age, 50 years; age range, 29 to 66 years) (see Table 1 for details).

**Table 1:** Intervertebral disc tissue sample details for mechanically stimulated annulus fibrosus cells

Sample	Sex	Mean age, yr	Disc level	Histological grade	Source
1	Male	57	L4/L5	1 (nondegenerative)	Postmortem
2	Male	46	L5/S1	1 (nondegenerative)	Postmortem
3	Male	37	L5/S1	1 (nondegenerative)	Postmortem
4	Male	57	L2/L3	7 (mildly degenerative)	Postmortem
5	Female	29	L4/L5	9 (degenerative)	Surgical (discectomy)
6	Male	66	L4/L5	9 (degenerative)	Surgical (discectomy)

### Immunohistochemical analysis of cytokine receptors in IVD cells

Paraffin-embedded AF and NP tissue obtained from a cohort of nine individuals, including patient and postmortem samples (mean age, 54 years; age range, 34 to 79 years) (Table 2), were processed for immunohistochemical analysis as previously reported [50,51], with articular cartilage tissue used as a positive control. Briefly, mounted sections were dewaxed in xylene and treated with trypsin (Invitrogen, Paisley, UK) for antigen retrieval, and endogenous peroxidase activity was blocked by using hydrogen peroxide (Fisher Scientific UK Ltd, Loughborough, UK). Samples were blocked with either 10% wt/vol rabbit serum with bovine serum albumin (BSA) (Sigma, Poole, UK) or 10% wt/vol donkey serum with BSA (Sigma, Poole, UK) and incubated at room temperature for 1 hour with primary antibodies for IL-4 receptor α (IL-4Rα) (1:300 dilution, catalogue no. MAB230; R&D Systems, Abingdon, UK), IL-2 receptor-γ (IL-2Rγ) (1:10 dilution, catalogue no. MAB284; R&D Systems, Abingdon, UK) or IL-13 receptor α_1 _(IL-13Rα_1_) (1:25 dilution, catalogue no. AF152; R&D Systems, Abingdon, UK). After being washed with Tris-buffered saline, samples were incubated at room temperature for 1 hour with biotinylated secondary antibodies (1:400 dilution of either rabbit anti-mouse, catalogue no. E0464; Dako, Ely, UK; or donkey anti-goat, catalogue no. SC-2042; Santa Cruz Biotechnology, Santa Cruz, CA, USA), and their binding was visualised using the streptavidin-biotin complex (Dako, Ely, UK) technique with 3,3'-diaminobenzidine tetrahydrochloride solution (Sigma, Poole, UK). Sections were counterstained with haematoxylin (Surgipath Europe Ltd, Peterborough, UK).

**Table 2:** Intervertebral disc tissue sample details based on immunohistochemical analysis

Sample	Sex	Mean age, yr	Disc level	Histological grade	Source
1	Male	37	L4/L5	1 (nondegenerative)	Postmortem
2	Male	61	L4/L5	2 (nondegenerative)	Postmortem
3	Male	30	L4/L5	2 (nondegenerative)	Postmortem
4	Male	61	L5/S1	5 (mildly degenerative)	Postmortem
5	Male	75	L4/L5	5 (mildly degenerative)	Postmortem
6	?	34	L5/S1	5/6 (mildly degenerative)	Surgical (discectomy)
7	Male	44	L4/L5	5 (mildly degenerative)	Surgical (discectomy)
8	Male	59	L4/L5	7 (mildly degenerative)	Postmortem
9	Male	79	L4/L5	7 (mildly degenerative)	Postmortem
10	Female	28	L4/L5	9 (degenerative)	Surgical (discectomy)

### Isolation and culture of AF cells

AF tissue was separated from the IVD within 24 hours of death or surgical removal and finely minced prior to enzymatic digestion as previously reported [18]. AF cells were cultured in standard medium (Dulbecco's modified Eagle's medium with 4.5 g/L glucose, GlutaMAX™ and pyruvate (Gibco, Invitrogen, Paisley, UK) containing 50 μg/mL ascorbic acid, 250 ng/mL amphotericin, 100 U/mL penicillin, 100 μg/mL streptomycin (Invitrogen) and 10% foetal calf serum (Invitrogen, Paisley, UK) and expanded in monolayers with medium changes every 2 or 3 days. Subconfluent AF cells with passage numbers ≤6 were trypsinised (Invitrogen, Paisley, UK), seeded onto untreated silicone membrane BioFlex culture plates (Flexcell International, Hillsborough, NC, USA) at a density of 1 × 10^5 ^cells/mL in 2 mL of standard medium and allowed to adhere for 48 hours (passage numbers >6 have been found to influence cell behaviour (J.A. Hoyland, unpublished data)). Media were changed to serum-free media 15 to 17 hours prior to the application of CTS.

### Application of CTS using the Flexcell Tension™ FX-4000™ System in the presence or absence of cytokine inhibitors

AF cells in serum-free media adhered to Bioflex culture plates were treated with or without the IL-1 receptor antagonist (IL-1Ra) (0.1 μg/mL) (catalogue no. 280-RA; R&D Systems) or a blocking antibody to the IL-4 receptor (IL-4RAb) (10 μg/mL) (catalogue no. MAB230; R&D Systems) 10 minutes prior to the application of CTS.

Using the Flexcell Tension™ FX-4000™ System, a CTS previously shown to alter AF cell function consisting of a 10% strain at 1.0-Hz frequency for 20 minutes was delivered to the base of the silicone membranes within the Bioflex culture plates, and consequently to the AF cells that adhered to these membranes, using computer-controlled negative pressure as previously described in detail [18]. The loading regime was chosen to be within the physiological range, with IVD tissue strains estimated to range from 1% to 25% during complex motions while compressed with a load physiologically similar to walking [52,53] and 1.0-Hz estimated to be the frequency of locomotion [54]. Unstimulated AF cells that adhered to Bioflex plates served as controls. Mechanically stimulated or unstimulated cytokine inhibitor-treated and untreated AF cells were incubated at 37°C with 5% CO_2 _for either 0 (baseline control), 1 hour (nondegenerative AF cells) or 24 hours (degenerative AF cells) postload, and total RNA was extracted. The time points for mRNA analysis were chosen on the basis of previously published observations. Of the time points investigated (0 (baseline control), 1, 3 or 24 hours after application of 1.0-Hz CTS), gene expression was altered at 1 and 24 hours in AF cells derived from nondegenerative and degenerative IVDs, respectively [18].

### Cell viability

Cell viability was assessed using the Trypan blue (0.4%; Sigma) exclusion assay as previously reported [18].

### Quantitative real-time PCR

Total RNA was extracted from each BioFlex culture plate well using TRIzol™ reagent (Invitrogen) according to the manufacturer's instructions, and samples were treated with DNase I (Ambion, Austin, TX, USA). RNA quality and quantity were determined using the Nanodrop ND-1000 Spectrophotometer (Nanodrop Technologies, Wilmington, DE, USA), and 500 ng of RNA were reverse-transcribed using the High Capacity 'cDNA' Reverse Transcription Kit (Applied Biosystems, Warrington, UK). A quantitative real-time polymerase chain reaction assay was performed in triplicate using TaqMan Universal PCR Master Mix (Applied Biosystems) with primers and probes for glyceraldehyde 3-phosphate dehydrogenase (*GAPDH*), aggrecan and type I collagen for degenerative AF cells, and *MMP3 *and a disintegrin and metalloproteinase with a thrombospondin type 1, motif 4 (*ADAMTS4*) for nondegenerative AF cells using previously published sequences and concentrations [18]. The genes were chosen for analysis on the basis of previously published observations whereby, from among a panel of genes investigated (aggrecan, types I and II collagen, *MMP3*, *MMP9*, *MMP13 *and *ADAMTS4*), only *MMP3 *and *ADAMTS4 *expression and aggrecan and type I collagen gene expression were altered in 1.0-Hz CTS-treated nondegenerative and degenerative AF cells, respectively [18]. Data were analysed using the 2^-ΔΔ*C*^_T _method [18,55] and normalised to the endogenous control gene *GAPDH *and unloaded baseline controls.

### Statistics

The nonparametric data as determined by the Shapiro-Wilke test were analysed using the Mann-Whitney *U *test.

## Results

### Immunohistochemistry

**Figure 1:**
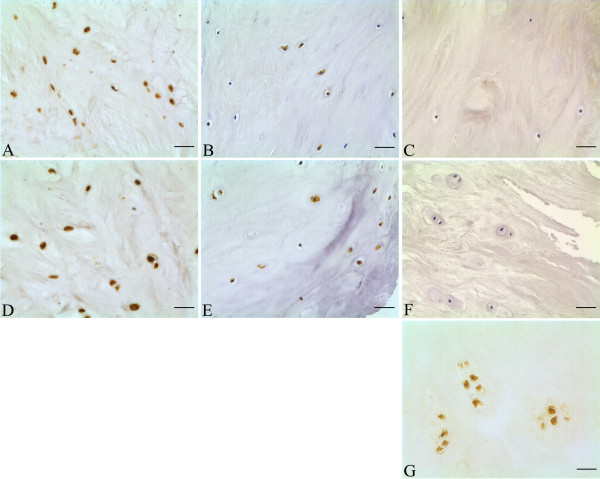
**Immunohistochemical studies showing the localisation of interleukin (IL)-4 receptor (IL-4R), IL-common γ receptor (IL-cγR) and IL-13 receptor (IL-13R) in the human intervertebral disc**. IL-4R immunopositivity in nondegenerative samples of **(a) **annulus fibrosus (AF) cells and **(d) **nucleus pulposus (NP) cells. IL-cγR immunopositivity in nondegenerative samples of **(b) **AF cells and **(e) **NP cells. IL-13R immunonegativity in nondegenerative samples of **(c) **AF cells and **(f) **NP cells. **(g) **A positive control slide is included for IL-13R in nondegenerative articular chondrocytes. Scale bar, 25 μm.

Immunopositivity was seen for IL-4R in both AF and NP cells from all nondegenerative and degenerative samples and was predominantly localised intracellularly throughout the cytoplasm and nucleus (Figures 1a and 1d). IL-2Rγ immunopositivity was also seen in both AF and NP cells from all nondegenerative and degenerative samples, with localisation occurring intracellularly but exclusively to the cytoplasm (Figures 1b and 1e). IL-13R immunopositivity was seen only in the positive control articular chondrocyte sample (Figure 1g), with no immunopositive cells in the AF or NP tissues of the nine samples investigated (Figures 1c and 1f).

### Mechanical stimulation of cytokine inhibitor-treated AF cells

AF cells isolated from nondegenerative and degenerative IVDs remained viable (>90%) throughout the culture period, with cell viability unaffected by either mechanical stimulation or cytokine inhibitor treatment. Unloaded controls showed no significant change in gene expression for any of the genes investigated at any of the time points analysed.

### 1.0-Hz CTS

#### Nondegenerative AF cells

**Figure 2:**
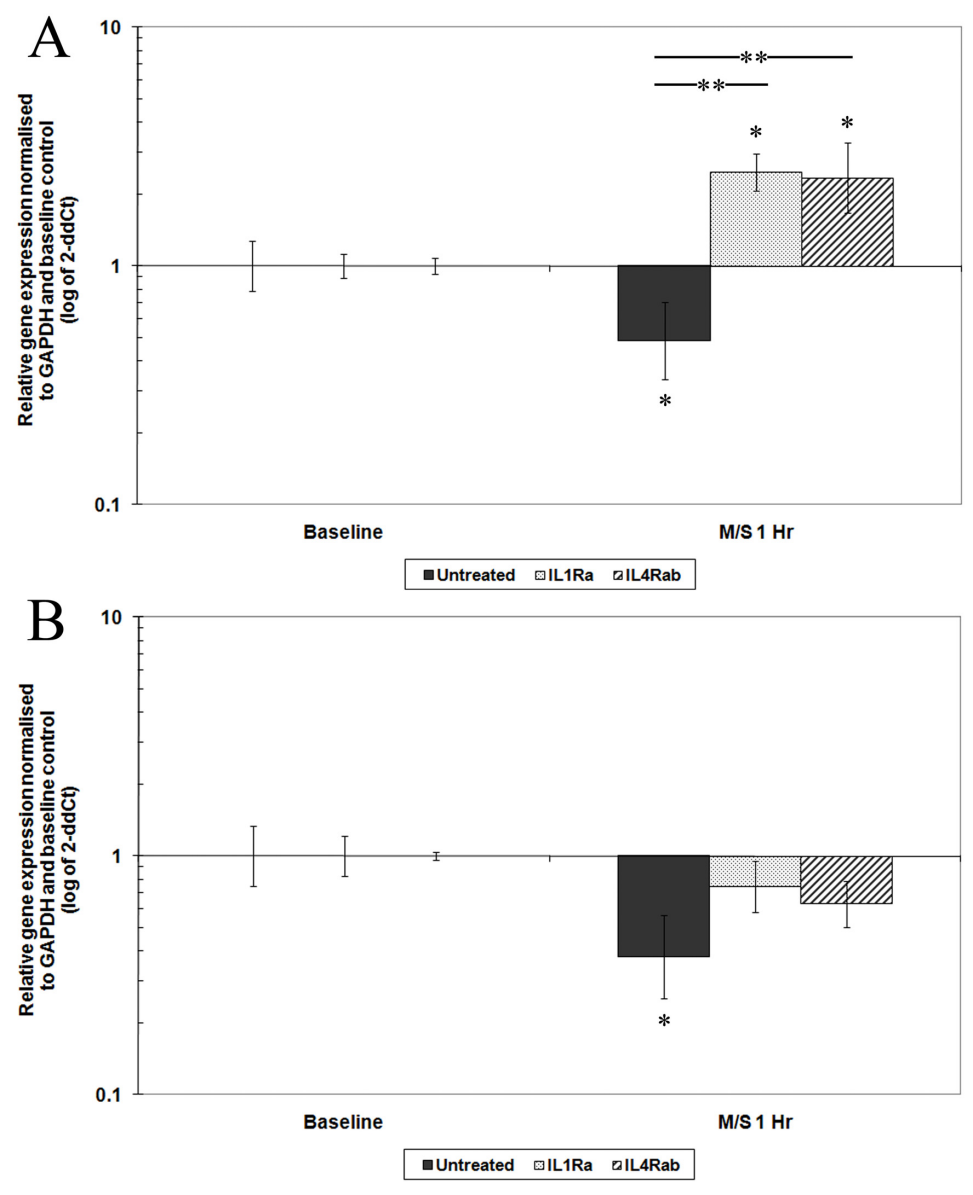
**Bar graphs showing the effect of cyclic tensile strain (CTS) on matrix-degrading enzyme gene expression of annulus fibrosus (AF) cells from nondegenerative intervertebral discs (IVDs) with or without cytokine inhibitors**. Cells derived from nondegenerative IVDs were treated with or without interleukin (IL)-1 receptor antagonist (IL-1Ra) (0.1 μg/ml) or IL-4 receptor antibody (IL-4RAb) (10 μg/ml) 10 minutes prior to mechanical stimulation with CTS at 10% strain at 1.0 Hz for 20 minutes, then incubated for 1 hour prior to analysis. A quantitative real-time polymerase chain reaction assay was used to analyse the gene expression of matrix-degrading enzymes **(a) **matrix metalloproteinase 3 and **(b) ***ADAMTS4 *relative to the housekeeping gene *GAPDH *(glyceraldehyde 3-phosphate dehydrogenase) and normalised to the corresponding unloaded baseline control. Black bars represent nondegenerative AF cells mechanically loaded without treatment, and the speckled bars and striped bars represent cells mechanically loaded after treatment with IL-1Ra or IL-4RAb, respectively. Values are means ± SEM; *n *= 3. *P ≤ 0.05 denotes a significantly significant difference in gene expression between mechanically stimulated (M/S) and unstimulated baseline controls. **P ≤ 0.05) denotes a significantly significant change in gene expression between mechanically stimulated baseline controls with or without cytokine inhibitors.

There was no change in the relative gene expression of aggrecan or type I collagen in the nondegenerative AF cells following 1.0-Hz CTS as previously reported [18], while *MMP3 *and *ADAMTS4 *were significantly decreased at 1 hour post-mechanical stimulation (sixfold, *P *< 0.05, and sevenfold, *P *< 0.05, respectively) (Figures 2a and 2b). Treatment of AF cells from nondegenerative IVDs with IL-1Ra 10 minutes prior to the application of 1.0-Hz CTS increased the baseline gene expression of *MMP3 *compared to untreated cells (threefold, *P *< 0.05). However, the application of load in the presence of IL-1Ra led to a further increase in *MMP3 *gene expression 1 hour post-CTS (twofold, *P *< 0.01), resulting in a significantly altered response compared to the load-induced decrease in *MMP3 *gene expression observed in untreated, mechanically stimulated cells (*P *< 0.01) (Figure 2a).

IL-4RAb treatment of AF cells derived from nondegenerative tissue had no effect on the baseline gene expression of *MMP3*. However, subsequent stimulation with CTS in the presence of IL-4RAb caused an increase in *MMP3 *gene expression compared to baseline (twofold, *P *< 0.01). Thus the load-induced decrease in *MMP3 *gene expression in untreated, mechanically stimulated cells was altered to an increase when pretreated with IL-4RAb (twofold, *P *< 0.01), with significance achieved between pretreated and untreated mechanically stimulated cells (*P *< 0.01) (Figure 2a). Incubation of nondegenerative AF cells with IL-1Ra or IL-4RAb prior to mechanical stimulation had no effect on the baseline gene expression of *ADAMTS4*. Pretreatment with either IL-1Ra or IL-4RAb followed by mechanical stimulation inhibited the load-induced decrease in *ADAMTS4 *gene expression compared to baseline, although this finding did not achieve statistical significance compared to the untreated loaded sample for both IL-1Ra- and IL-4RAb-treated cells (Figure 2b).

#### Degenerative AF cells

**Figure 3:**
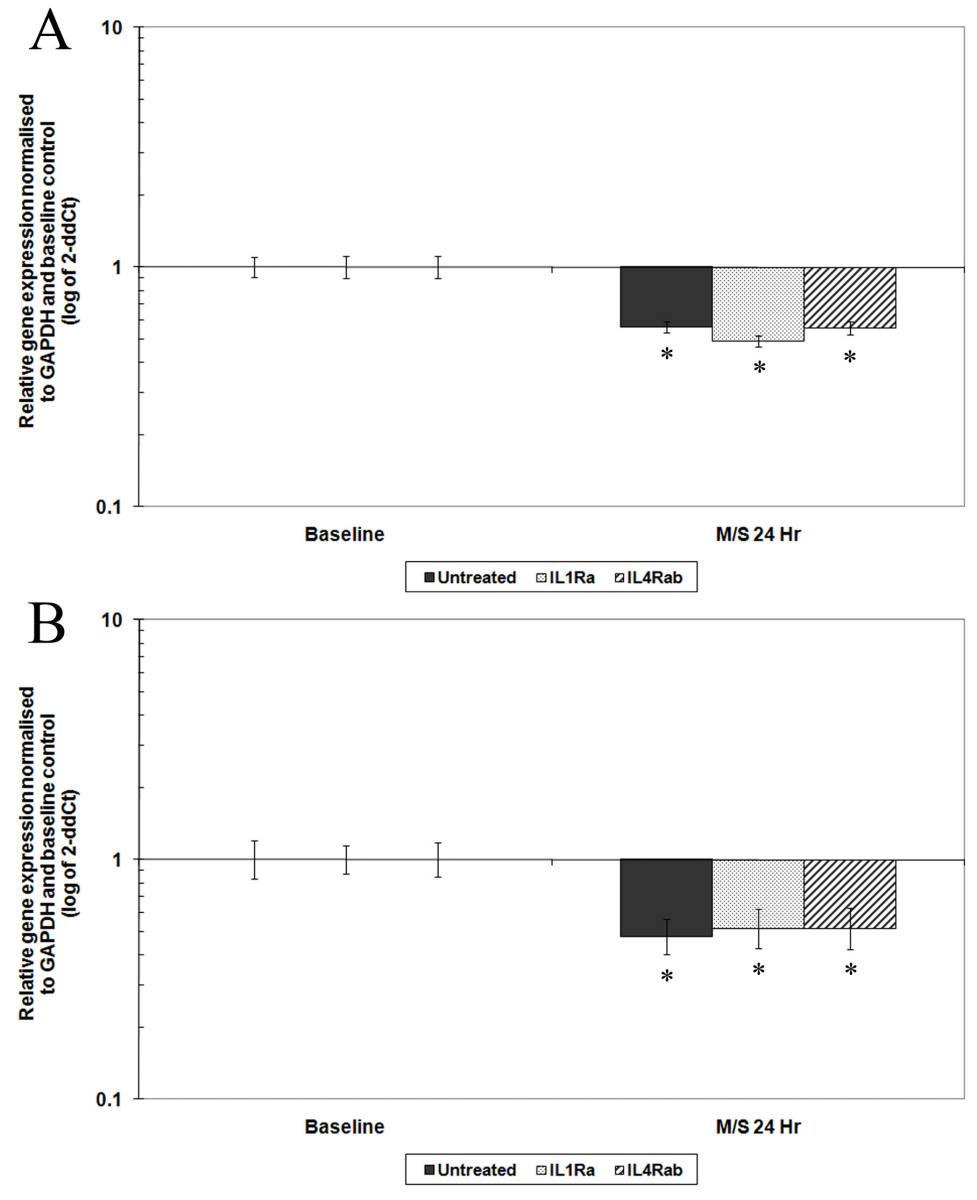
**Bar graphs showing the effect of cyclic tensile strain (CTS) on matrix protein gene expression of annulus fibrosus (AF) cells from degenerative intervertebral discs (IVDs) with or without cytokine inhibitors**. Cells derived from degenerative IVDs were treated with or without interleukin (IL)-1 receptor antagonist (IL-1Ra) (0.1 μg/ml) or IL-4 receptor antibody (IL-4RAb) (10 μg/ml) 10 minutes prior to mechanical stimulation with CTS at 10% strain and 1.0 Hz for 20 minutes, then incubated for 24 hours prior to analysis. A quantitative real-time polymerase chain reaction assay was used to analyse the gene expression of matrix proteins **(a) **aggrecan and **(b) **type I collagen relative to the housekeeping gene *GAPDH *(glyceraldehyde 3-phosphate dehydrogenase) and normalised to the corresponding unloaded baseline control. Black bars represent degenerative AF cells mechanically loaded without treatment, while speckled and striped bars represent cells mechanically loaded after treatment with IL1-Ra or IL4-RAb, respectively. Values are means ± SEM; *n *= 3. *P ≤ 0.05 denotes a statistically significant change in gene expression between mechanically stimulated (M/S) and unstimulated baseline controls.

Mechanical stimulation of AF cells derived from degenerative IVDs with 10% strain at 1.0-Hz frequency for 20 minutes resulted in a significant decrease in aggrecan and type I collagen relative gene expression 24 hours post-CTS (fivefold, *P *< 0.01, and sixfold, *P *< 0.05, respectively) (Figures 3a and 3b). There was no change in the relative gene expression of *MMP3 *or *ADAMTS4 *in degenerative AF cells stimulated with 1.0-Hz CTS, as previously reported [18]. Treatment of degenerative AF cells with IL-1Ra or IL-4RAb prior to mechanical stimulation had no effect on the baseline gene expression level of aggrecan and no effect on the CTS-induced decrease in aggrecan gene expression, with aggrecan gene expression remaining significantly decreased (sixfold, *P *< 0.01, and fivefold, *P *< 0.05, for IL-1Ra-and IL-4RAb-pretreated mechanically stimulated cells, respectively) (Figure 3a). Treatment of degenerative AF cells with IL-1Ra or IL-4RAb caused a decrease in baseline type I collagen gene expression (threefold, *P *< 0.01, and fourfold, *P *< 0.01, respectively). However, pretreatment with either IL-1Ra or IL-4RAb did not inhibit the load-induced decrease observed in type I collagen gene expression at 24 hours post-CTS, with type I collagen gene expression showing a further decrease compared to pretreated, unstimulated cells (sixfold, *P *< 0.01, and sixfold, *P *< 0.01, for IL-1Ra- and IL-4RAb-pretreated mechanically stimulated cells, respectively) (Figure 3b).

## Discussion

Mechanical stimulation in the form of CTS is important in controlling AF cell matrix homeostasis, with the degree of disc degeneration influencing the mechanoresponse of AF cells. However, to date, the molecular signalling pathways enabling AF cells to sense and respond to mechanical stimuli, a mechanism termed 'mechanotransduction', remain to be elucidated. The role of cytokines as mechanotransducers has been reported in a range of cell types, including endothelial cells [37], bone cells [34,35], tenocytes [56-58] and chondrocytes [25,26,40]. However, to our knowledge, this is the first study to investigate the involvement of IL-1 and IL-4 in IVD, specifically AF cell mechanotransduction, as well as the first study to ascertain whether their involvement differs with degeneration.

### 1.0-Hz nondegenerative tissue

When stimulated at 1.0-Hz CTS, AF cells derived from nondegenerative tissue responded with a decrease in *MMP3 *and *ADAMTS4 *relative gene expression, suggesting a shift towards a less catabolic phenotype. This reduced catabolic response following 1.0-Hz CTS appears to be IL-1- and IL-4-dependent, as treatment with the cytokine inhibitors IL-1Ra or IL-4RAb inhibited the load-induced decrease in *ADAMTS4 *gene expression and caused an increase in *MMP3 *gene expression.

Unexpectedly, treatment of AF cells derived from nondegenerative tissue with IL-1Ra caused an increase in the baseline level of *MMP3 *gene expression prior to load. MMP activity has previously been shown to be inhibited following treatment of IVD tissue with IL-1Ra [59], suggesting that the unexpected IL-1Ra-induced increase in *MMP3 *gene expression observed in this study might correlate negatively with enzyme activity. This potential phenomenon should be investigated further because increased *MMP *gene expression in IVD cells caused by inhibited enzyme activity due to IL-1Ra treatment could pose a concern with regard to IL-1Ra as a potential treatment for DDD. Although enzyme activity may be regarded as the more biologically significant factor, elevated levels of MMP mRNA (with the potential for protein translation) following IL-1Ra treatment could be detrimental to IVD tissue homeostasis.

Previous studies have reported modulation of increased IL-1 β-dependent *MMP *gene expression following treatment with IL-1Ra [60]; however, the response of *MMP *gene expression to IL-1Ra treatment in the absence of IL-1 agonist has never been reported until now. Our data indicate that, in the absence of an agonist, treatment with IL-1Ra upregulates *MMP3 *gene expression by a currently undefined mechanism. It is also worth noting that treatment with IL-4RAb had no effect on the baseline gene expression level of *MMP3 *in AF cells derived from nondegenerative tissue, suggesting that the maintenance of baseline *MMP3 *gene expression occurs independently of IL-4. Importantly, although baseline *MMP3 *gene expression was upregulated following treatment with IL-1Ra, exposure of the cells to 1.0-Hz CTS caused a further significant increase in gene expression. This finding is opposite to that observed in untreated mechanically stimulated cells in which CTS caused a decrease in *MMP3 *gene expression. Such results implicate IL-1 in the CTS-induced decreased catabolic response observed in nondegenerative AF cells.

Treatment of AF cells derived from nondegenerative tissue with either cytokine inhibitor IL-1Ra or IL-4RAb had no effect on the baseline gene expression level of *ADAMTS4*, suggesting that the maintenance of *ADAMTS4 *baseline gene expression occurs independently of both IL-1 and IL-4. This observation is in contrast to IL-1Ra-dependent *MMP3 *baseline gene expression. However, the CTS-induced downregulation of *ADAMTS4 *gene expression in AF cells derived from nondegenerative tissue does appear to be cytokine-dependent, with cytokine inhibitors of both IL-1 and IL-4 preventing the decrease in gene expression following mechanical stimulation.

Although the expression of IL-4 has previously been reported in the IVD [46], expression of its receptors has not yet been described. Here we report, for the first time, and albeit in a small number of samples, immunopositivity for the IL-4 receptor subunits IL-4Rα and IL-2Rγ and immunonegativity for the receptor subunit IL-13Rα_1 _in human IVD cells. During IL-4R activation, IL-4 first binds IL-4Rα, leading to the recruitment of a second receptor subunit, predominantly IL-2Rγ in cells of haematopoietic origin [61] (termed 'type I IL-4R') and IL-13Rα_1 _in cells of nonhaematopoietic origin [62-64] (termed 'type II IL-4R'). It is therefore surprising that IL-4 appears to signal through type I IL-4R (IL-4Rα/IL-2Rγ heterodimer) in human IVD cells and not through type II IL-4R (IL-4Rα/IL-13Rα_1 _heterodimer) as reported in other cartilaginous tissues such as articular cartilage [65]. The downstream signalling pathways activated during IL-4 signalling are IL-4R type-dependent, with differential Janus kinase (JAK) phosphorylation occurring between activated receptor types (phosphorylated JAK1/3 [66,67] and JAK2/tyrosine kinase 2 (Tyk2) [65,68,69] following IL-4R types I and II activation, respectively) suggesting that IL-4-dependent mechanotransduction could differ between IVD cells and cells from other cartilaginous tissues.

In addition to IL-4, IL-1 also appears to be necessary for human AF cell mechanotransduction following 1.0-Hz CTS, with similar alterations and inhibitions to the CTS-induced decreases in *MMP3 *and *ADAMTS4 *gene expression, respectively. IL-1 β has previously been shown to be involved in human bone cell mechanotransduction, where an autocrine/paracrine IL-1 β-induced release of prostaglandin E_2 _is suggested to precede the 0.33-Hz CTS-induced hyperpolarisation response [35]. IL-1 β has also been implicated in osteoarthritic (but not nonosteoarthritic) chondrocyte mechanotransduction, where the pretreatment of cells with neutralising antibodies to IL-1 β prevented the CTS-induced depolarisation response, suggesting altered mechanotransduction in chondrocytes derived from degenerative tissue [40]. Although in our study it is not clear which subtype of IL-1 is involved in AF cell mechanotransduction, information from other studies of connective tissue mechanical loading suggests IL-1 β as the primary candidate [35,40]. Although the involvement of IL-1 in disc cell mechanotransduction has not previously been reported, the expression of IL-1 (IL-1α and IL-1 β), its antagonist (IL-1Ra) and its receptor (IL-1 receptor, type I) have been reported in both nondegenerative and degenerative IVDs, with the IL-1 agonists, but not the IL-1 antagonist, increasing with the severity of degeneration [42]. Furthermore, evidence from a study using rats suggests that potential interactions exist between the IL-1 and CTS signalling pathways in AF cells as demonstrated by the partial inhibition of IL-1 β-induced catabolic gene expression with the addition of 6% CTS treatment [44]. Thus, in addition to the role of IL-1 as a catabolic factor implicated in DDD, our findings support the concept that it may play a role in transducing physiological mechanical stimuli leading to tissue remodelling.

### 1.0-Hz degenerative tissue: an altered mechanotransduction pathway with disease

When AF cells derived from degenerative tissue were subjected to 1.0-Hz CTS, the observed response, reduced relative gene expression of the matrix proteins aggrecan and type I collagen, did not appear to involve either IL-1 or IL-4 as demonstrated by the inability of the cytokine inhibitors IL-1Ra and IL-4RAb to prevent the CTS-induced changes in gene expression. Although pretreatment with either cytokine inhibitor had no effect on the baseline gene expression level of aggrecan in AF cells derived from degenerative tissue, type I collagen baseline gene expression was reduced in these cells. This reduced type I collagen baseline gene expression might be expected following IL-4Rab treatment, as inhibition of chondroprotective IL-4 could be predicted to have a reduced anabolic or even catabolic effect. However, the fact that reduced type I collagen baseline gene expression followed treatment with IL-1Ra is an unexpected observation. These data therefore suggest that treatment of human AF cells derived from both nondegenerative and degenerative tissue with cytokine inhibitors appears to have a different effect from that previously observed in the presence of agonists.

Although treatment with either cytokine inhibitor caused a decrease in type I collagen baseline gene expression, exposure of the cells to 1.0-Hz CTS led to a further decrease in gene expression, suggesting that while IL-1 may be involved in basal collagen expression, neither IL-1 nor IL-4 is necessary for this mechanotransduction response. This lack of cytokine-dependent mechanotransduction was also observed in the regulation of aggrecan, where treatment of degenerative AF cells with either cytokine inhibitor IL-1Ra or IL-4RAb had no effect on baseline or 1.0-Hz CTS-induced downregulation of aggrecan gene expression.

Anabolic and catabolic gene expression is known to occur through the involvement of different transcription factors (for example, SRY (sex-determining region Y)-box 9, or SOX9, and nuclear factor κ-light chain enhancer of activated B cells (NFκB) for matrix protein and matrix-degrading enzyme gene regulation, respectively), with specific transcription factors regulated via specific signalling pathways (for example, IL-1-dependent NFκB regulation). Thus, differences in the regulation of matrix protein and matrix-degrading enzyme gene expression between AF cells derived from degenerative and nondegenerative tissue, respectively, could be due to differences in cytokine-dependent transcription factor activation brought about by the differential cytokine involvement observed with 1.0-Hz CTS between nondegenerative and degenerative AF cells.

This lack of involvement of IL-1 and IL-4 in the mechanoresponse of degenerative cells indicates that an altered mechanotransduction pathway may be in operation. Although cells from osteoarthritic cartilage have also been shown to signal through an altered mechanotransduction pathway in response to CTS [40], the alteration in signalling occurred through the involvement of an additional cytokine, namely IL-1β, and not through the loss of cytokine signalling shown to be necessary for mechanotransduction to occur in the absence of disease as reported here. These changes in signalling pathways indicate further differences in mechanobiology between these cartilaginous cell types.

Interestingly, NP cells derived from degenerative tissue have also been shown to utilise an altered mechanotransduction pathway following stimulation with HP. Le Maitre *et al. *[29] reported that the HP-induced decrease in aggrecan gene expression occurred via an RGD-recognising integrin pathway in NP cells derived from nondegenerative, but not degenerative, tissue, suggesting that disc cell mechanotransduction becomes altered with degeneration, which is in agreement with the results of this study.

## Conclusions

In conclusion, this study has investigated the involvement of the cytokines IL-1 and IL-4 in the mechanotransduction pathways of human AF cells derived from nondegenerative and degenerative tissue following exposure to a stimulus previously shown to alter matrix-regulating gene expression. We identified that the altered mechanoresponse observed in AF cells derived from degenerative tissue exposed to a CTS of 10% strain and 1.0-Hz frequency [18] may be the result of an altered mechanotransduction pathway. Studying the effect of CTS on human AF cells *in vitro *simplifies the complexity of *in vivo *loading, enabling uncharacterised intrinsic factors to be removed from the system. Although this nonphysiological loading environment has limitations in terms of relevance to the *in vivo *situation, removal of these uncharacterised biomechanical and biochemical factors enables the effect of cell deformation (and inevitably fluid flow) to be considered in isolation, allowing a more specific mechanotransduction pathway to be investigated. Mechanical forces are now well-recognised as important regulatory factors in IVD cell biology, and differences in the responses of cells from diseased discs are being reported. To date, the mechanisms involved in enabling IVD cells to respond to mechanical stimuli (and the mechanisms preventing a "normal" response by cells of diseased discs) remain largely unknown, demonstrating a requirement for the continued elucidation of IVD cell mechanotransduction, with the potential for the discovery of novel therapeutic targets which could lead to the prevention and/or treatment of DDD.

## Abbreviations

ADAMTS4: a disintegrin and metalloproteinase with a thrombospondin type 1 motif 4; AF: annulus fibrosus; BSA: bovine serum albumin; CTS: cyclic tensile strain; DDD: degenerative disc disease; ECM: extracellular matrix; GAPDH: glyceraldehyde 3-phosphate dehydrogenase; HP: hydrostatic pressure; Hz: hertz; IL: interleukin; IVD: intervertebral disc; JAK: Janus kinase; MAPK: mitogen-activated protein kinase; MMP: matrix metalloproteinase; NFκB: nuclear factor κ-light chain enhancer of activated B cells; NO: nitric oxide; NP: nucleus pulposus; PKC: protein kinase C; qRT-PCR: quantitative real-time polymerase chain reaction; R: receptor; Ra: receptor antagonist; RAb: receptor antibody; RNA: ribonucleic acid; SOX9: SRY (sex-determining region Y)-box 9; Tyk2: tyrosine kinase 2.

## Competing interests

The authors declare that they have no competing interests.

## Authors' contributions

HTG participated in the study design; performed all cell culture experimentation, molecular studies and data analysis; and drafted the manuscript. JAH helped to conceive the study and secure funding, participated in the study design and coordination, analysed the results and co-wrote the manuscript. AJF participated in the study design and coordination and was responsible for the grading of tissue samples. SJM conceived the study, secured funding, participated in its design and coordination, analysed the results and co-wrote the manuscript. All authors read and approved the final manuscript.
